# Suprapapillary trisectoral deployment of slim fully covered metal stents with ultra-stiff high-sliding guidewires for malignant hilar biliary obstruction

**DOI:** 10.1055/a-2452-5261

**Published:** 2024-11-13

**Authors:** Tadahisa Inoue, Rena Kitano, Tomoya Kitada, Kazumasa Sakamoto, Satoshi Kimoto, Jun Arai, Kiyoaki Ito

**Affiliations:** 1 12703 Department of Gastroenterology, Aichi Medical University, Nagakute, Japan

Placement of bilateral suprapapillary uncovered metal stents is recommended for unresectable malignant hilar biliary obstruction (MHBO) owing to superior patency compared with plastic stents, unilateral placement, and positioning across the papilla 1 2 3 . Recent studies also suggest that bilateral trisectoral placement further improves survival, especially in patients undergoing chemotherapy 4 . However, stent occlusion still affects approximately half of all cases, and reintervention for occlusion is technically demanding after bilateral placement of uncovered metal stents. Therefore, we proposed a novel approach, suprapapillary trisectoral placement of a slim, fully covered metal stent, which reduces the risk of side-branch occlusion and is easily removable, facilitating straightforward reintervention. Although insertion of the second and third stents becomes primarily challenging with this method, utilizing an ultra-stiff, high-sliding guidewire can circumvent this issue ( Fig. 1 ).

**Fig. 1:**
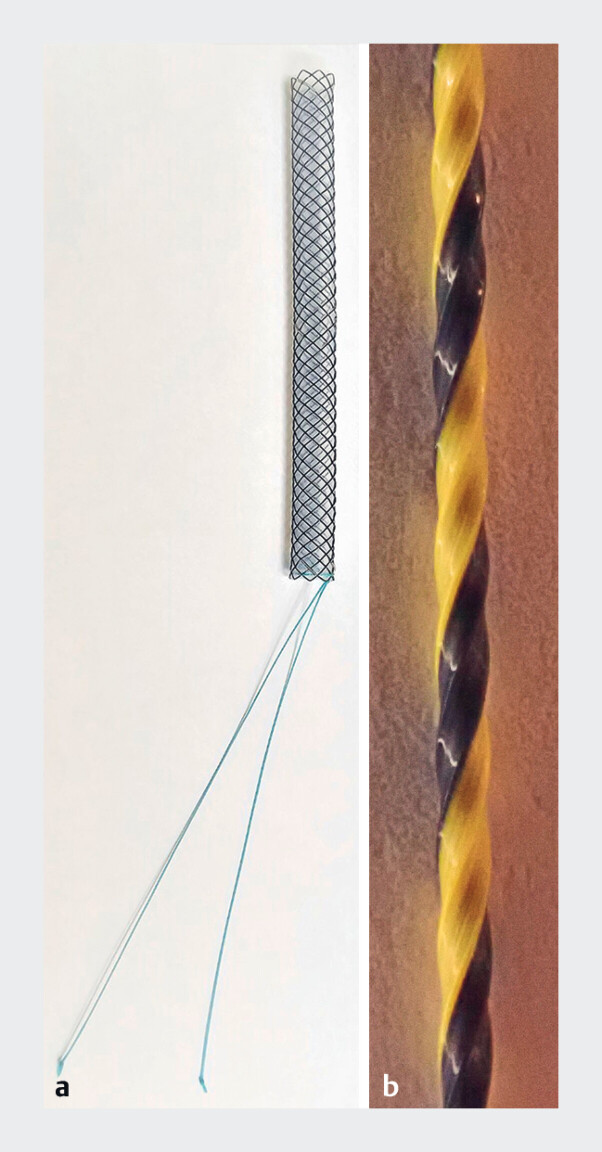
Device images. **a** The slim, fully covered metal stent (Taewoong Medical, Seoul, Korea) has a diameter of 6 mm, incorporates a retrieval string at the distal end, and boasts a 7.5-Fr delivery system. **b** The novel 0.035-inch guidewire (SeekMaster Hard; Piolax Medical Devices, Kanagawa, Japan) has a thick, 0.7-mm, high-rigidity, nickel-titanium core with polytetrafluoroethylene coating that undergoes “ridge-processing” to minimize contact area and friction. This process improves the deliverability and trackability of the entire system.

The metal stent is 6 mm in diameter, incorporates a retrieval string at the distal end, and boasts a 7.5-Fr delivery system. The novel 0.035-inch guidewire features a 0.7-mm high-rigidity, nickel-titanium core with polytetrafluoroethylene coating. Notably, this coat undergoes “ridge-processing” to minimize contact area and friction, thereby improving the deliverability and trackability of the entire system.

An 88-year-old man presented with obstructive jaundice due to Bismuth type IIIa MHBO. After placing three novel guidewires into the right posterior, right anterior, and left hepatic ducts, respectively, the delivery system of the stent was introduced over the left guidewire and deployed across the stricture and above the papilla. The second metal stent was subsequently inserted over the posterior guidewire and deployed alongside the left metal stent. Finally, the third metal stent was introduced over the anterior guidewire and deployed in a stent-by-stent configuration ( Fig. 2 , Video 1 ). All three retrieval strings were positioned through the duodenal papilla. No adverse events or stent dysfunction occurred until the patient’s death.

**Fig. 2:**
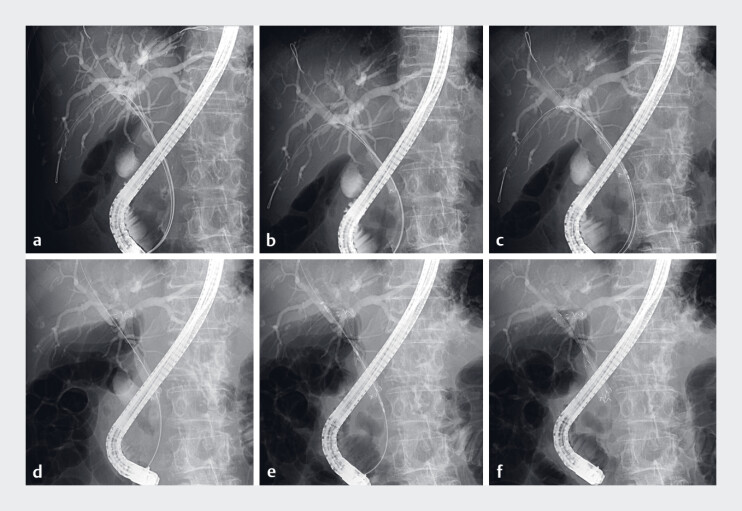
Fluoroscopic images. **a** Three novel, ultra-stiff, high-sliding guidewires were placed into the right posterior, right anterior, and left hepatic ducts, respectively. **b** The delivery system of the first slim, fully covered metal stent was introduced over the left guidewire and deployed across the stricture and above the papilla. **c** The second metal stent was then inserted over the posterior guidewire. **d** The second stent was deployed alongside the left metal stent. **e** Finally, the third metal stent was inserted over the anterior guidewire. **f** The third stent was deployed in a stent-by-stent configuration.

Suprapapillary trisectoral deployment of slim, fully covered metal stents with ultra-stiff, high-sliding guidewires for malignant hilar biliary obstruction.Video 1

Overall, suprapapillary trisectoral placement of slim, fully covered metal stents shows potential as an uncomplicated and advantageous procedure for MHBO.

Endoscopy_UCTN_Code_TTT_1AR_2AZ

## Notes

Endoscopy E-Videos https://eref.thieme.de/e-videos
*E-Videos* is an open access online section of the journal *Endoscopy* , reporting on interesting cases and new techniques in gastroenterological endoscopy. All papers include a high-quality video and are published with a Creative Commons CC-BY license. Endoscopy E-Videos qualify for HINARI discounts and waivers and eligibility is automatically checked during the submission process. We grant 100% waivers to articles whose corresponding authors are based in Group A countries and 50% waivers to those who are based in Group B countries as classified by Research4Life (see: https://www.research4life.org/access/eligibility/ ). This section has its own submission website at https://mc.manuscriptcentral.com/e-videos .
